# Proteomic Technology “Lens” for Epithelial-Mesenchymal Transition Process Identification in Oncology

**DOI:** 10.1155/2019/3565970

**Published:** 2019-10-29

**Authors:** Monica Neagu, Carolina Constantin, Marinela Bostan, Constantin Caruntu, Simona Rebeca Ignat, Sorina Dinescu, Marieta Costache

**Affiliations:** ^1^Department of Immunology, Victor Babes National Institute of Pathology, Bucharest 050096, Romania; ^2^Department of Pathology, Colentina University Hospital, Bucharest 020125, Romania; ^3^Faculty of Biology, University of Bucharest, Bucharest 76201, Romania; ^4^Immunology Center, “Stefan Nicolau” Virusology Institute, Bucharest 030304, Romania; ^5^Carol Davila University of Medicine and Pharmacy, Bucharest 050474, Romania; ^6^Department of Dermatology, Prof. N.C. Paulescu National Institute of Diabetes, Nutrition and Metabolic Diseases, Bucharest 011233, Romania

## Abstract

The epithelial-mesenchymal transition (EMT) is a complex transformation process that induces local and distant progression of many malignant tumours. Due to its complex array of proteins that are dynamically over-/underexpressed during this process, proteomic technologies gained their place in the EMT research in the last years. Proteomics has identified new molecular pathways of this process and brought important insights to develop new therapy targets. Various proteomic tools and multiple combinations were developed in this area. Out of the proteomic technology armentarium, mass spectrometry and array technologies are the most used approaches. The main characteristics of the proteomic technology used in this domain are high throughput and detection of minute concentration in small samples. We present herein, using various proteomic technologies, the identification in cancer cell lines and in tumour tissue EMT-related proteins, proteins that are involved in the activation of different cellular pathways. Proteomics has brought besides standard EMT markers (e.g., cell-cell adhesion proteins and transcription factors) other future potential markers for improving diagnosis, monitoring evolution, and developing new therapy targets. Future will increase the proteomic role in clinical investigation and validation of EMT-related biomarkers.

## 1. Introduction

The epithelial-to-mesenchymal transition (EMT) process allows the cellular differentiation from polarized epithelial phenotype to mesenchymal characteristics. Also an active process in embryogenesis and wound healing, EMT plays a decisive role in cancer development being highly coordinated at genetic, epigenetic, and proteome levels by different regulators [1].

**Figure 1:**
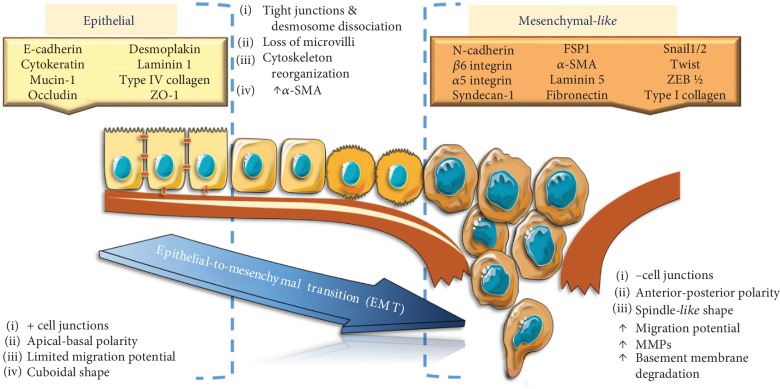
Markers and main molecular changes during epithelial-to-mesenchymal transition.

The EMT process is marked by loss of epithelial marker E-cadherin, induced by the upregulation of certain transcriptional repressors (e.g., SNAIL1/2, TWIST, and ZEB1/2). As the EMT process occurs, the epithelial cells acquire multiple cell-surface and cytoskeletal markers, as well as expression of certain extracellular proteins and transcription factors [2, 3] (Figure 1). Thus, gaining a set of mesenchymal markers supports and stabilizes the newly acquired cellular phenotype. One of the main changes in the expression of cell-surface markers is the cadherin switch, from E-cadherin high expression in epithelial cells to its low profile correlated with an increased expression of N-cadherin in mesenchymal cells [4]. EMT also alters cell-extracellular matrix (ECM) interactions, and as a result, integrins such as *β*6 integrin, *α*5 integrin, and syndecan-1 could also be used as biomarkers for EMT, depending on the type of cancer [5]. In terms of cytoskeletal markers, the expression of fibroblast-specific protein 1 (FSP1), vimentin, *α*-smooth muscle actin (*α*-SMA) correlated with low expression or loss of cytokeratins, mucin-1, occludin, and desmoplakin is an indicator of cells undergoing EMT [6]. Another cytoskeletal marker, *β*-catenin, can be a marker of either epithelial normal cells or mesenchymal cells, depending on its cell localization [5].

In addition, FOXO proteins modulate EMT process during tumour development events contributing to the design of additional studies for future development of FOXO proteins as therapeutic targets [7].

Regarding EMT initiation, there are still debates whether metabolic changes are contributory or just an effect of EMT triggering. Catabolic and anabolic pathways that characterize glucids, amino acids, and lipid metabolism are equally involved. Depending on the tumour cell type, the EMT is based on an aerobic glycolytic program and unveils a lipogenic metabolism depending on the EMT inducer (e.g., TNF-*α* versus TGF-*β*-derived Snail1). However, all these metabolic events lead to membrane fluidity increase and destabilization of lipid rafts. This would upregulate membrane cholesterol content making inhibitors of cholesterol synthesis to act for stabilizing lipid rafts, reducing membrane fluidity, and therefore opposing EMT phenomena [8]. An overview of the protein profiling of the EMT process is depicted in Figure 1.

In the very last years, proteomic strategies were enrolled for analysing different milestones in ETM course, real powerful tools for catching and quantifying ETM marks starting from the cellular surface to intracellular events. The proteomic domain comprises a large array of technologies, from traditional ELISA to newer ones, but all of them gaining their place in oncology investigations. MS, with its various variants, like matrix-assisted laser desorption/ionization (MALDI) and electrospray, was subject for Nobel prizes [9]. Soon enough, this proteomic technology was assisted by increased automation and computational analytics software. These last MS's acquired traits have pushed the technology in clinical proteomics [10]. More recently, multiple reaction monitoring was introduced using triple quad MS technology and/or several tribrid high-resolution MS. The evolution of these new types of MS was a consequence of the clinical proteomic needs seeking to measure detailed proteomic panels further to be used in early detection, disease and/or therapy monitoring, patient stratification, and so on [11]. Another proteomic technology that is highly used in clinical approaches is protein microarray (PM) technology with all its functional versions like multiplexed, tissue arrays, direct, and reverse phase [12]. Combinations of various array technologies can now not only show that the protein is deregulated in terms of concentration and structure but also show where this protein is located in a diseased tissue [13]. Hence, from identifying and validating myriads of biomarkers to identifying complex proteomic networks involved in tumourigenesis [14], PM has accelerated clinical proteomics, offering new insights in disease and therapy monitoring.

Tumourigenesis is such a complex process involving a myriad of more subtle events like chronic inflammation and viral infection [15–20]; thus, the proteins that are up-/downregulated in this complex process can be in minute concentrations at one time point. Thus, besides evaluating extremely small amounts of a biological sample for a huge panel of proteins, their actual quantities are in minute concentrations, some of them probably just a few hundred per cell. In clinical proteomics, samples contain small amounts of cells, and if the sample is just a biopsy and not an excised tumour, the sample is even smaller with actually a few hundred to a thousand cells. Invented more than 15 years ago, the reverse phase protein array (RPPA) has been used broadly in medicine [13]. Therefore, RPPA was developed to measure proteins and their phosphorylated forms that exist in low levels in small biopsy samples [12].

Through a time-resolved multiomic strategy, a correlation between proteome changes, phosphoproteome signalling, and histone alterations during TGF-*β*-induced EMT was recently highlighted. Thus, Erk signalling activation and histone H3K27me3 posttranslational modification were the most significantly upregulated marks in TGF-*β*-induced EMT that could be turned in therapeutic strategies for EMT-related diseases [21].

Using antibody-based protein profiling with matching transcriptomic data, it was defined an EMT signature consisting of 239 genes in an analysis comprising 736 cancer cell lines. Thus, histone deacetylase inhibitors as inducers of EMT and kinase inhibitors as mesenchymal-to-epithelial transition promoters were the most prevailing markers uncovered [22]. The EMT is linked with and promotes cancer stem cell (CSC) formation and mediates drug resistance, making tumour microenvironment a promising source of clinical markers and/or therapeutic targets in cancer [23].

EMT candidate markers that may help to diagnose diseases or monitor treatment efficiently could be detected *via* proteomic tools. One of the most engaged technologies is mass spectrometry- (MS-) based platforms that allow analysing multiple analytes and samples and moreover allow differentiation between epithelial and mesenchymal tumours and in addition could depict how these can be assessed for future target discovery and validation [24], contributing for better understanding of the physiological and pathological bases in EMT process [25].

Even more, an intricate process like ETM would beneficiate from the latest proteomic approaches to study the dynamics of this complex process.

## 2. EMT Proteomics

### 2.1. Cellular Models in EMT Proteomics

Although many studies regarding EMT rely on the significant role that endothelial cells (EC) play in this crucial process of tumour invasiveness and migration [2], the EMT connection with tumourigenesis and metastatic course was initially unveiled in various epithelial cancer cell line models [26]. Furthermore, EMT in tumourigenesis is a demanding task as certain molecular similarities are shared by human tumour cells with mesenchymal phenotype and stromal fibroblasts [27]. Therefore, such studies using different cancer cell line models provided data regarding the relevance of EMT to the metastatic process [28]. Moreover, this challenge is nowadays transposed in studies involving human tumour samples to highlight the clinical role of the EMT process [26, 29].

The interference of EMT with tumour cell fate is studied in various *in vitro* models of cancers focusing mainly on cellular morphology in a definite *omic* context generated by tumour cell transformation. The myriad of factors released in tumour milieu are seized nowadays with high-throughput proteomics (MS, 2D electrophoresis, arrays platforms, etc.) that further decipher those specific alterations imprinted in the tumour milieu.

#### 2.1.1. Breast and Genital Cancer

Factors released by EC generate alterations specific for EMT and moreover could tip the balance toward EMT or toward MET. Thus, in a model with human breast cancer SK-BR-3 cells exposed to EC supernatants, it was revealed by quantitative MS proteomics that *nidogen-1* inhibits SK-BR-3 cell migration. This nidogen-1 is exclusively derived from confluent EC and reveals a novel pathway of cancer progression controlled by EC [2].

Proteomic analysis is able to decode fine molecular mechanisms of EMT triggering. For example, SNAIL is one of the transcription factors overexpressed in EMT and is directly correlated with cancer invasiveness [30]. Overexpression of SNAIL in breast adenocarcinoma cell line MCF7 was validated by molecular and functional proteomic tests. Comprehensive proteomic subcellular fractionation corroborated with GEL-LC-MS/MS revealed 4,289 intracellular proteins involved in cell cycle and epigenetic control. This complex protein network analysis show that SNAIL overexpression led to cell cycle arrest in G0/G1. Moreover, SNAIL upregulation is favoured by HDAC1 inhibition suggesting an interrelation between these two proteins in EMT initiation. These molecular mechanisms underlying EMT might also represent effective strategies for cancer metastasis management [31]. Another modulator of EMT, TWIST, is a highly conserved transcription factor that also acts as a major regulator of EMT; however, little is known about signalling regulation by TWIST in cancer cells. By iTRAQ labelling combined with 2D LC-MS/MS, 194 proteins mainly involved in MAPK, PI3K/AKT, and WNT signalling were identified with a highly modified expression in Twist-overexpressing MCF10A mammary epithelial cells. Ingenuity Pathway Analysis showed that ITGB1 integrin interconnects ILK kinase and FAK kinase as well as MAPK, PI3K/AKT, and WNT pathways. Amplified TWIST and ITGB1 expressions are associated with tumour progression and EMT in MCF10A cells. Inactivating ILK, FAK, MAPK, or PI3K/AKT signalling would also suppress EMT, so the ITGB1-FAK/ILK axis revealed by proteomic inquires conducts the TWIST-induced EMT in human breast cancer cells [32].

More recently, breast cancer metastasis was associated with alterations in genes encoding for complex I components of mitochondria. A quantitative proteomic analysis in a model with highly metastatic MDA-MB-231 cells has assessed certain proteins encoded by these genes and pointed out that it could have significant value in EMT. Using the iTRAQ labelling, it was found that NDUFB9, one of the respiratory complex I subunits, was downregulated; moreover, NDUFB9 knockdown cells MDA-MB-231 exhibit decreased expression of E-cadherin and an increased expression of vimentin and fibronectin-1, as assessed by immunoblotting analysis. Downregulation of NDUFB9 activates Akt/mTOR signalling pathway, which led to EMT endowing MDA-MB-231 cells with a metastatic phenotype. Thus, complex I mitochondrial deficiency could be a potential biomarker for assessing EMT process with possible clinical utility [33]. Another study model with MDA-MB-231 cells was used to assess the transition from a nontumourigenic epithelial-like phenotype to an aggressive mesenchymal-like one. HMGA1, a nonhistone chromatin protein, is a key regulator of EMT process. The proteins isolated from HMGA1-silenced MDA-MB-231 cells were analysed using label-free shotgun MS. Data obtained generated an HMGA1 protein signature of 21 members endorsed with prognostic value in breast cancer. Further, qRT-PCR, Western blot, and immunohistochemistry validated the association of three proteins (KIFC1, LRRC59, and TRIP13) with HMGA1 expression levels and with tumour cell dissemination. Besides their potential as a druggable target, these factors aid in elucidating EMT in a triple-negative breast tumour [34].

EMT process is a very appropriate barograph in assessing therapeutic effects of certain drug agents in breast cancer. Thus, the effect of two retinoic acid isomers was examined in MDA-MB-231 human breast cancer cell line. Bottom-up proteomic strategies (2D SDS-PAGE, MALDI-TOF/TOF) were applied for identifying more than 50 proteins affected by retinoic acid isomers, from which 9 proteins are associated with the tumour process. Exposure to retinoic acid isoforms led to a decreasing of the protein level related to cellular metabolism, apoptosis, and regulation of the transcription process or EMT, namely, annexins, nucleoside diphosphate kinase B, and vimentin [35]. EMT process could guide the therapy options in cancer cells expressing oncogenic Ras mutants. An MS-based method using KRasG12V-transfected MCF10A (MCF10A-KRasG12V) cells were used for molecular profiling of the cell membrane proteins. This combined proteomic scrutiny identified over 500 cell-surface proteins deregulated in MCF10A-KRasG12V cells that depict as a real proteomic map the phenotypic changes consistent with EMT process and further uncovers potential therapeutic targets [36]. Intercellular communication in EMT is another topic of interest. Recent data involving proteomic assessment demonstrated that the protein cargo of exosomes reflects the *epithelial*/*mesenchymal phenotype* of secreting breast cancer cells. Thus, breast cancer phenotypes may be differentiated based on their protein content carried by exosomes [37].

In endometrioid carcinoma (ENC), it was reported previously that aldehyde dehydrogenase 1 (ALDH1), a potential marker of normal and malignant stem cells, is related to the tumourigenic potential. Using shotgun proteomics, the levels of several proteins were compared in human ENC cells with high and low ALDH1 expressions; it was noticed that *serum deprivation-response protein* (SDPR) was particularly expressed in cells with high ALDH1 expression. SDPR is a protein required for the formation of caveolae. By means of SDPR-knockout ENC cells generated with CRISPR/Cas9 tool, it was shown that SDPR was correlated with invasion, migration, and EMT [38].

At distant sites, the interaction of circulating tumour cells (CTCs) with the microenvironment is crucial for metastatic colonization, with the participation of the extracellular vesicles (EVs). Tumour EVs replicate the *epithelial* phenotype predominant in the primary carcinoma, whereas CTCs are regarded as *EMT* phenotype. The epithelial-like EVs were characterized using *SILAC proteome analysis* in Hec1A endometrial cell line model. There was an *in vitro* indication of improved adhesion of CTC to a functionalized endothelium, suggesting a contribution of the epithelial-like EVs in the homing of CTCs at metastatic sites and indirectly in EMT commencement [39].

Approaches with proteins map in EMT were extended to ovarian cancer where three cell lines—the cell line-derived ovarian cancer stem cells (OCSCs), 3AO, and Caov3—were subjected to proteome pattern surveying using *liquid chromatography*- (*LC*-) *MS*/*MS label*-*free quantitative proteomics*. Over 70 proteins were found most differentially expressed, and among them, stonin 2 (STON2) was suggested to downregulate the stemness of the ovarian cancer cell which is characterized by EMT-related markers. The identified protein network revealed that STON2 modulate stemness in ovarian cancer cells *via* epigenetic effectors such as DNMT1, and therefore, STON2 has a role in ovarian cancer biology and could represent a therapeutic target [40].

Oct4A is a well-known biomarker for cancer stem cells, and recently, its key roles were revealed in ovarian tumour cell survival, metastasis, and drug resistance. An MS-based proteomic analysis performed in ovarian cancer shRNA Oct4A knockdown cell line uncovers important alterations in protein networks related to cytoskeleton, ECM, cell proliferation, drug resistance, and EMT, sustaining Oct4A role in modulating EMT related to ovarian tumours [41].

#### 2.1.2. Renal Carcinoma

Multiomic platforms were enrolled also in exploring EMT in renal carcinoma through modulators of transcription factors such as SNAIL, well known to be involved in EMT initiation [2]. Thus, BRCA1-associated protein 1 (BAP1) is an enzyme from the ubiquitinase family whose encoding gene BAP1 is mutated in almost 10% of clear cell renal cell carcinomas (ccRCC). Lower BAP1 expression is related to prolonged overall survival; quantitative proteomics of BAP1 knockout ccRCC cell lines revealed a decreased expression of transcriptional repressor SNAIL and reduced activity of Rho-GTPase events that uphold EMT process [42].

#### 2.1.3. Melanoma

In melanoma, EMT process is related to some transcription factors that affect cellular physiology. For instance, a higher expression of the osteogenic master gene RUNX2 has been reported in melanoma cells associated to tumour progression and EMT. In a melanoma cell model with RUNT-deleted cells by CRISPR/Cas9 technique, a reduced cell proliferation, increased apoptosis, and reduced EMT traits, indicating RUNX2 as a likely therapeutic target [43], were reported.

To another scale, phenotype switching toward more aggressive forms is related to EMT. The NK cells may increase the malignancy of melanoma cells by inducing changes relevant to EMT that are reliant on NKp30 or NKG2D receptors and a concomitant IFN*γ* and TNF*α* release. Melanoma cells suffering EMT either increase their HLA-I surface expression or inhibit tumour-recognizing activating receptors thus avoiding the NK cell attack. In several different melanoma cell lines derived from metastatic melanoma resections, MS analysis revealed proteomic profiles induced by coculture with NK cells or by EMT cytokines; it was observed a partial overlapping in proteomic pattern depending on the milieu exposure (NK cells or EMT factors) that could be exploited in innovative antitumour therapies NK cell-based [44].

#### 2.1.4. Lung Cancer

In lung cancer cells, functional proteomic screening was enrolled to assess EMT via Raf-MEK-ERK pathway modulation by KAP1. In A549 lung cancer cells, it was found that knockdown KAP1 arrested cells in the G0/G1 phase and decreased growth, metastasis, and EMT process; thus, Raf-MEK-ERK pathway represents a source of therapeutic inquires and regulate lung cancer development [45].

#### 2.1.5. Digestive Tumours

A key step in hepatocellular carcinoma (HCC) development is the migration of malignant hepatocytes into blood vessels that would sustain the spreading of HCC tumour cells. This transendothelial migration depends on TGF-*β* which promotes EMT that would favour HCC cell spreading. In a model of hepato-transendothelial migration using EMT-transformed hepatocytes (MIM-RT) and liver sinusoidal endothelial cells (mLSECs) [46, 47], specific molecular changes in both migrating hepatocytes and endothelial cells were detected by MS. There was reported a proteome pattern comprising 36 and 559 regulated proteins in hepatocytes and endothelial cells, respectively, suggesting that transendothelial migration also depends on intercellular interactions and not on TGF-*β* only. There were identified alterations on peroxiredoxin-3, epoxide hydrolase, transgelin-2, and collectin 12, markers associated clinically with patient's survival. This hepatocellular plasticity sustained by an EMT phenotype induced by TGF-*β* provides valuable clues in HCC invasion mechanisms [48].

EMT regulation is also ensued at the epigenetic level, so UHRF1 maintains the optimum level of DNMT1-mediated DNA methylation, being involved in various tumour processes. Recently, it was shown that UHRF2 has a role in EMT process regulation by acting as a coregulator of the EMT-transcription factors (TFs). The following human gastric cancer cell lines—SGC7901, MKN74, N87, and MKN45—with ectopically expressed UHRF2 were subjected to proteome profiling that has revealed upregulation of many EMT-TFs in UHRF2-overexpressing cells. Moreover, by ChIP-seq, it was identified that UHRF2 and EMT-TFs share the same genomic binding motifs, and the interactome analysis highlights that UHRF2 interact with TFs (e.g., TCF7L2), proteins involved in chromatin remodelling and histone alterations, data confirmed by immunoprecipitation combined with MS. Giving this evidence from multidimensional proteomic analysis, a role of UHRF2 in transcription coregulator for EMT and metastasis mechanisms was proposed [49].

#### 2.1.6. Other Cancers

The differential action of TGF*β*, known as EMT inducer on cancer-associated fibroblasts (CAFs) and on epithelial tumour cells (ETCs), has been recently investigated at the proteomic level. In a coculture system comprising fluorescently labelled CAFs and ETCs stimulated with TGF*β*, cells were separated using FACS and subjected to quantitative MS. It was shown that TGF*β* treatment upregulates extracellular matrix proteins and increased N-cadherin levels in CAFs, whereas ETCs were found low responders to TGF*β*. The authors conclude that TGF*β* treatment could change proteome pattern in fibroblasts and epithelial tumour cells and thus modulate EMT phenotype [50].

A similar approach involving quantitative MS combined with gene arrays was used in NCI-H226 mesothelioma cells to assess EMT pathway in the frame of BAP1 tumour suppressor activity influence. Analysed proteome and gene expression revealed enrichment in proteins related to cytoskeleton as well as enhancement in markers related to EMT. Further functional evaluation in BAP1 wild-type, BAP1 knocked down, and BAP1 noncatalytically expressing NCI-H226 mesothelioma cells indicate that BAP1 enzymatic activity was a requisite to maintain these proteomic and genomic phenotypes [51].

A complex proteomic approach corroborated with qPCR target validation was used to identify differentially expressed proteins in a coculture of human glioma U251 cells treated with human bone marrow mesenchymal stem cells (hBMSCs). It was registered that hBMSCs could inhibit cell proliferation and could induce apoptosis of U251 cells. By means of proteomics, there were identified 11 differentially expressed proteins involved in biological processes mostly related to the PI3K/AKT pathway. In addition, hBMSC treatment led to inhibition of EMT-like and PI3K/AKT pathways. These proteomic data highlight the antitumour properties and EMT inhibitors of hBMSCs with the potential to be explored in glioma therapy [52].

### 2.2. EMT Markers in Tumour Tissue—Proteomic Approaches

#### 2.2.1. Digestive Tract Tumours

*Colorectal cancer* (*CRC*) actually represents the most studied solid cancer for EMT proteomic analysis, and various proteomic technologies applied in patient's samples were employed to study the complexity of EMT process underlying CRC. The worldwide high incidence of CRC and its metastatic capacity leading to disabilities and unfortunately death streamline the proteomic assessment that has revealed a high array of deregulated proteins in tumour samples that were found linked to EMT. Thus, recently, using laser capture microdissection, CRC tissue samples were collected and further subjected to iTRAQ-based quantitative proteomic analysis to evaluate global proteomic profiling of CRC tumour microenvironment. Comparing samples of tumour microenvironment vascular endothelial cells (VECs) from patients subjected to antiangiogenic therapy, a large array of differentially expressed proteins was identified in VEC, comprising over 200 different protein types. The majority of these proteins were proven to be involved in EMT process, ECM-receptors, focal adhesion, PI3K-Akt signalling pathway, angiogenesis, and HIF-1 signalling pathways. Owing to this large proteomic profiling, future targets related to EMT could be developed in CRC [53].

The cellular heterogeneity of tumour microenvironment with various clones of cancer cells, stroma cells, cancer stem cells, and immune cells represents a rich reservoir for clinical meanings in EMT process. In over 400 CRC samples, Xu et al. investigated various EMT markers along with immune cell markers investigated. Protein expression of certain stemness markers (e.g., Nanog, Lgr5, and CD44v6) and infiltrating immune cells were highly correlated; furthermore, all these markers positively correlated with E-cadherin or Snail. Authors even denominated a protein cluster (SIE) that could predict 5-year survival of CRC patients. This cluster is comprised of proteins that are related to cancer stemness, immune status, and EMT process [54].

Using high-resolution proteomic technologies, namely, nano-LC-MS/MS coupled to Orbitrap mass spectrometry, liver metastases of CRC revealing that the ECM is deregulated by cancer cell-derived peptidyl-arginine deiminase 4 (PAD4) were evaluated. Citrullination of collagen type I as a key component of ECM is involved in the EMT promotion and liver metastasis, showing PAD4 involvement in the progression of CRC to metastasis [55].

The study of functional proteome in over 250 CRC tumour samples that were further compared to over 450 samples from The Cancer Genome Atlas (TCGA) has identified various protein patterns. The 163 validated proteins were accomplished with RPPA. This large study pointed out that proteins that append to the EMT pattern characterized a subtype A while high Akt/TSC/mTOR pathway characterized subtype B. Prognostic relevance of these patterns was analysed, and the authors concluded that eight proteins could predict tumour recurrence, and these proteins were collagen VI, FOXO3a, INPP4B, LcK, phospho-PEA15, phospho-PRAS40, Rad51, and phospho-S6 [56].

After developing genomic studies, proteomic confirmations have established that high-mobility gene group A2 (HMGA2) has as direct downstream target IL11 that modulates cell migration through pSTAT3-dependent signalling pathway. Therefore, in over 120 CRC sample tissues, a strong positive correlation was found between HMGA2 and IL11 expression and further was associated with poor prognosis and with other clinical parameters like tumour size and lymph node tumour invasion. Thus, Wu et al. pointed out that HMGA2 and IL11 can be new therapy targets in CRC [57].

In a recent report, Zhang et al. have studied in CRC samples that prostate transmembrane protein androgen induced 1 (PMEPA1) is linked to EMT. Gene expression microarray and further immunoproteomics indicate that PMEPA1 is highly expressed in tumours compared to normal tissue and is associated with a poor prognosis. EMT process is promoted by PMEPA1 through the activation of bone morphogenetic protein (BMP) signalling through TGF-*β* action [58].

Integrin-linked kinase (ILK) was also studied in relation to CRC progression and chemoresistance. In around 150 tumour samples, it was shown through immunohistochemical analysis that ILK expression correlates with EMT and CSC markers. Moreover, this overexpression is associated with metastasis and chemoresistance [59]. In a larger cohort comprising over 400 CRC samples, immunohistochemistry showed a particular S100A8+ cell type harboured in the tumoural stroma that was found associated with EMT markers, like E-cadherin and SNAIL, and moreover, this association could predict CRC prognosis [60].

Using proteomic and genomic technologies aided by bioanalysis, several EMT biomarkers were identified in CRC samples (e.g., BGN, MMP1, LGALS1, SERPINB5, and TM4SF4) and appended to the pathway of TGF*β*/Snail triggered with TNF*α*/NF*κ*B. The authors point out that poor prognosis is associated with these biomarkers that are reported as being involved in EMT process [61].

Lipocalin2 (LCN2) expression in 400 CRC samples was studied with various proteomic technologies involving immunohistochemistry and Western blot and linked to EMT process. LCN2 was found highly expressed in over 60% of the tumour samples, and it was found significantly correlated with the presence of E-cadherin in the membrane and with the absence of nuclear *β*-catenin. The authors point out that LCN2 can be a negative regulator of EMT in CRC, acting upstream of NF-*κ*B/snail signalling network. Thus, therapeutical manipulation of LCN2 and NF-*κ*B/snail pathway can be a future approach in CRC [62].

Interleukin-13 (IL-13) can be another candidate molecule triggering EMT. Thus, by Western blot and immunoblot analysis, it was reported in CRC samples a positive correlation between IL-13R*α*1 and ZEB1 probably demonstrating that IL-13/IL-13R*α*1/STAT6/ZEB1 axes promote EMT and aggressiveness of this cancer [63].

Growth differentiation factor 15 (GDF15) can enhance EMT process *via* TGF-*β* receptor that further activates Smad2 and Smad3 pathways. Indeed, in CRC tissues, GDF15 is overexpressed and was correlated with its increased serum levels in patients diagnosed with CRC. A high level of GDF15 prognosticated reduced overall survival in CRC. GDF15 activates EMT and thus can be considered a new prognostic marker [64].

In a study involving highly invasive colon cancer cell lines and CRC samples, correlations between Cdc42BPA overexpression and clinic-pathological patient parameters were found. Using tissue microarray, it was found that Cdc42BPA expression is higher in CRC samples when compared to adjacent normal tissues. Cdc42BPA expression correlated with metastasis and worse prognosis [65].

*Gastric cancer* (*GC*) is a very heterogeneous disease characterized by a high rate of dissemination; thus, studies that focus on the EMT process seek to evaluate pathways that can prognosticate the disease outcome and new therapy approaches. In GC, the FGFR1 expression was reported positively correlated with SNAI1, VIM, and ZEB1 expression but negatively correlated with CDH1. Furthermore, FGFR1 expression was associated with peritoneal dissemination of the tumour and with EMT that was reflected in poor prognosis for GC patients [66]. Recent proteomic reports have shown that GC comprises two molecular subtypes, the mesenchymal (MP) and epithelial (EP) phenotypes. While MP subtype is associated with poor prognosis and high resistance to chemotherapy, EP subtype induces better survival rates and sensitivity to chemotherapy. Integrative proteomic analysis has revealed proteins involved in the EMT-related pathways and insulin-like growth factor 1 (IGF1)/IGF1 receptor (IGF1R) pathway. With this newly acquired knowledge regarding these pathways, novel therapeutic targets can become eligible and developed in GC [67].

In HCC, the metastasis process is correlated with subtle processes like GnT-V-mediated N-glycosylation of marker CD147/basigin. This is actually a tumour-associated glycoprotein upregulated when EMT process is triggered *via* TGF-*β*1 activation. Moreover, GnT-V expression is controlled by PI3K/Akt pathway so that this recent study provided new evidence for developing specific drugs that can impede metastasis [68]. In HCC tissues, proteomic studies were performed to investigate the differences between early recurrence and late recurrence cases. 2D fluorescence gel electrophoresis was used as the main proteomic technology, and after investigating over 1,600 proteins, 19 proteins were selected based on their capacity to differentiate between these two groups of patients. Transglutaminase 2 (TGM2) was found upregulated in the early recurrence patients, and TGM2's mRNA level was correlated with EMT-related genes. This study has shown, once more, that proteomic evaluations in HCCs can be further developed for identifying new therapeutic targets in metastatic HCC [69]. Another study has enrolled quantitative proteomics and Ingenuity Pathway Analysis to prove that TGM2 overexpression in HCC is correlated with inflammatory signalling pathways. Moreover, the promotion of EMT process in HCC was shown to be mediated by pseudohypoxia triggered by TGM2/VHL/HIF-1a pathway [70].

In primary liver carcinoma, a proteomic retrospective study has shown that a novel peptide containing the RGD (Arg-Gly-Asp)-sequence derived from the C-terminal portion of fibrinogen was detected in the sera of metastatic patients. This peptide was associated to neoangiogenesis and EMT process [71].

#### 2.2.2. Breast and Genital Cancers

In terms of applying proteomic technologies to depict EMT process, the second most studied cancer is breast cancer (BC). Like in CRC, breast cancer has a high incidence and, at the molecular level, is characterized by various subtypes. In the search to evaluate complex proteomic biomarkers in BC, various proteomic technologies were recently applied. Liquid chromatography-selected reaction monitoring MS (LC-SRM) was reported to aid the oncology domain because it can quantify multiple biomarkers. In BC, the American Society of Clinical Oncology (ASCO) has validated some tissue markers for evaluating prognosis and guiding therapy, like estrogen receptor, progesterone receptor, and HER2/Neu receptor tyrosine kinase. LC-SRM technology assessed these proteins and their phosphorylation status, and furthermore, these proteins correlated with Ki-67 (proliferation marker) and vimentin (tumour aggressiveness markers) as related to EMT process. Within this study, Chen et al. reported the design of a three-tier multiplexed assay platform, for evaluating the complex biology of BC tissues [72].

Like in other solid cancers, tumour ECM has an important role in BC tumourigenesis. Using an array of proteomic approaches (2D differential gel electrophoresis, MALDI-MS, and immunoblotting), the deregulated expression of ECM proteins was studied. There is a specific ECM pattern characterized by a series of dysregulated proteins, e.g., fibrinogen-*β* chain, collagen *α*-1(VI) chain, and *α*-1B-glycoprotein. In triple-negative BC that displays the mentioned ECM pattern, there is an increase of FGG and *α*5*β*1/*α*v*β*3 integrins accompanied by a decrease of detyrosinated *α*-tubulin and mimecan. These deregulated expressions induce integrin disorganization involving focal adhesion kinase and activation of EMT-related Rho GTPases. This proteomic profiling in BC could be developed in prognosis biomarkers [73]. Inside triple-negative BC, there is a subset of claudin-low (CLOW) type. The Clinical Proteomic Tumour Analysis Consortium (CPTAC) has recently reported a proteogenomic evaluation of this BC CLOW type. Dihydropyrimidinase-like-3 (DPYSL3) protein was found specific for CLOW subset, and it was suggested that DPYSL3 constitutes a key protein in the negative feedback for EMT. Thus, through this protein, CLOW tumours can be identified as being sensitive to PAK signalling inhibitors during EMT process [74].

In BC, fibroblasts trigger the activation of breast cancer stem cells (BCSCs), while CAFs induce EMT and therefore favour a stem cell profile. Cells isolated from BC samples, namely, normal fibroblasts (NFs) and CAFs, subjected to proteomic analyses have revealed some interesting findings. Proteomics has shown that these cells are heterogeneous and can trigger BCSC generation, with an emphasis on CAF potency. Thus, CAFs induced aldehyde dehydrogenase-1-positive (ALDH1+) BCSCs, while NFs generated mostly CD44+CD24-type BCSCs [75].

Using a newly established flow cytometry surface proteomics combined with cellular functional analysis, several characteristics of metastatic breast cancer (MBrCa) explants were studied. On these cells, several markers were found upregulated: CD200, CD51/CD61 (from the integrin *α*5/*β*3 family), CD26 (dipeptidyl peptidase-4), CD165 (c-Cbl), and CD54 (ICAM-1). When EMT process evolves and it is accompanied by invasion, a series of proteins are once more upregulated (e.g., CD26, CD63 (LAMP3), CD105 (Endoglin), CD107a (LAMP1), CD108 (Semaphorin 7A), CD109 (Integrin *β*4), CD151 (Raph blood group), and disialoganglioside G2). When comparing these data to standard breast cancer cell lines (MDA-MB-231, MCF7, and BT-474), the authors have shown that MBrCa have a clear mesenchymal pattern and that surface proteome is different when compared to standard BC cell lines [76].

Basal-like breast cancer (BLBC) is a type of BC associated with poor prognosis. Using several genomic and proteomic technologies, aldo-keto reductase 1 member B1 (AKR1B1) was found overexpressed in BLBC. The authors show a positive feedback loop where Twist2 induces the transcription of AKR1B1 activating nuclear factor *κ*B (NF-*κ*B) that further upregulates Twist2. Thus, this positive loop will activate EMT complex process. Moreover, epalrestat, AKR1B1 inhibitor, could suppress CSC characteristics in BLBC tumours [77].

Invasive lobular carcinoma (ILC) is a subtype of histological BC, having the second occurrence after invasive ductal carcinoma (IDC). In a broad genomic, proteomic (e.g., RPPA), transcriptomic, and clinical data performed on ILC, Michaut et al. reported subtyping ILCs. Thus, ILC has an “immune-related subtype” characterized by upregulation of PD-L1, PD-1, and CTLA-4 and higher sensitivity to DNA-damaging cytostatics and a “hormone-related subtype”, which is related to EMT and several genomic traits (e.g., gain of chromosomes 1q and 8q and loss of chromosome 11q) [78]. Previously reported by other groups [79], the study of Michaut et al. confirmed that ILCs can beneficiate from new therapeutical agents as PI3K pathway inhibitors [78].

In the class of genital cancers, high-grade serous ovarian cancer (HGSOC) has a high recurrence rate, mainly due to the high rate of drug resistance. In an established human ovarian carcinoma cell line, isolated from a patient with chemorefractory HGSOC, complex characterization was performed using genomic, transcriptomic, and proteomic technologies like MS. High levels of alpha-enolase and vimentin were found characterizing an EMT profile of this cell line [80]. In ovarian cancer, EGFR family of protein overexpression depicts an aggressive behaviour. In an ovarian adenocarcinoma cell line (Caov-3), the inducement of EMT was studied using subcellular proteome enrichment, GEL-LC-MS/MS, and SILAC platform. Signalling pathways like PI3K/Akt/mTOR and Ras/Erk MAPK were found activated when EMT was induced with EGF in this Caov-3. The study shows that EGF-induced EMT in ovarian cancer cells induces also deregulations in protein synthesis, cell cycle control, CSC generation, and the clinical poor prognosis of the patients [81].

In cervical cancer cells, another protein with enzyme function, O-GlcNAc transferase (OGT), was proven to be involved in EMT regulation. Gao et al. described in 2018 for the first time OGT-interacting proteins, like PRMT5/WDR77 complex, PRC2 complex, the ten-eleven translocation (TET) family, CRL4B complex, and nucleosome remodelling and deacetylase (NuRD) complex. OGT upregulation in cervical cancer was shown to be related to worse prognosis. The study pointed out that OGT is related to EMT and can be both prognosis biomarker and future target therapy [82].

Using immunoprecipitation and MS-based quantitative proteomic approaches in a long established cervical cancer cell line (HeLa), ADAM12 was reported to interact with various proteins. Among these proteins, myoferlin was reported as regulating ADAM12 expression, reducing its specific substrate, E-cadherin. All these mechanistic links regulate cell adhesion and metastasis [83].

#### 2.2.3. Other Cancers

There are several sparse studies that have used proteomic analysis to evaluate EMT process in the metastatic outcome of cancers.

*(1) Head and Neck Cancers*. In head and neck squamous cell carcinoma (HNSCC), recurrence has a high incidence, most probably due to minimal residual disease (MRD). In a complex research flow involving also membrane proteomic methodology [84], several interesting protein expressions were found. Thus, EGFR was found highly expressed and moreover found constitutively phosphorylated; CD10, a marker for CSC, was as well found overexpressed. Therefore, Roh et al. show that in HNSCC, markers appending to EMT process can be hallmarks of recurrence and they can be further developed for testing MRD in these patients [85]. In human oral squamous cell carcinomas (OSCCs), nucleosome remodelling and deacetylase (NuRD) complex regulates tumourigenesis processes, and the loss of the subunit Deleted in Oral Cancer 1 (DOC1) associates with protumourigenesis and EMT processes. When restoring DOC1 in OSCC cells, EMT process is reversed through a regulatory process in which SWI/SNF and NURD have antagonistic functions that control chromatin and transcription [86]. Using RPPA profiling in nasopharyngeal carcinoma (NPC), total protein expression and protein functions were analysed in correlation to metastasis. In NPC, metastasis was associated with proteins regulating signalling pathways that control the cell cycle, apoptosis, and EMT [87].

*(2) Renal Cancers*. Renal cell carcinoma (RCC) is characterized by a high degree of metastasis. Zhou et al., using a proteomic approach, have shown that in tumour tissues, MYSM-1 is downregulated. Tissue microarray has shown that the low expression is associated with poor clinical prognosis. Inducing overexpression of MYSM-1 suppressed cell proliferation, migration, and invasion, inhibiting EMT process [88]. In clear cell renal cell carcinoma (ccRCC), an integrated proteomic and transcriptomic evaluation has shown that in all metastasis stages of ccRCC, the most differentially expressed molecules were TGF-*β* and proteins related to EMT. From this complex process, serpin peptidase inhibitor clade H member 1 (SERPINH1) was found strongly associated with poor clinical prognosis. These proteins related to EMT could stratify ccRCC patients that need a more aggressive therapeutical approach [89]. In urothelial carcinoma (UC), MS and quantitative proteomics were used for investigating in a patient's urine biomarkers associated to EMT. Shotgun proteomics identified over 200 candidate proteins in which signalling pathways of SH3 domain binding glutamic acid-rich protein like 3 (SH3BGRL3) were the most prominent one. SH3BGRL3 expression associates with risk of progression for UC patients. SH3BGRL3 is involved in EMT process promotion and cell migration. Thus, evaluation of urinary SH3BGRL3 can identify a subset of patients that need a more aggressive treatment in order to impede the disease progression [90].

## 3. EMT Highlights in Therapy

### 3.1. New Therapy Targets

As already stated prior, there are several types of solid cancers, e.g., BC and digestive cancers, which are taking advantages on proteomic approaches seeking for new therapy targets.

#### 3.1.1. Breast Cancer

In BC, proteomic studies have been performed using quantitative multiplexed proteomic tandem mass tags (TMTs) to address new therapy targets. Hence, in a recent study, TACC3 (transforming acidic coiled-coil protein 3) inhibitor, KHS101, suppresses cancer cell stemness and EMT processes and induces apoptosis. Upon applying this proteomic analysis, multiple protumoural processes were shown to be hindered by this inhibitor; thus, KHS101 can be foreseen as a multitargeting inhibitor in BC [91]. Among the genes that characterize BC cells that develop EMT or its reverse process MET, those that encode for proteins involved in DNA replication and repair pathways, ABC transporter, Hedgehog, Notch, and several metabolic pathways were found deregulated, and these can be also future therapy targets [92].

In a BC cell line model using several proteomic methods, new drugs were screened to abolish EMT processes. Thus, a combination of resveratrol (RSVL) with salinomycin (SAL) showed good efficacy in these cell lines. Western blots, colony formation, and flow cytometry for cellular apoptosis were used to show that EMT-associated proteins (e.g., fibronectin, vimentin, N-cadherin, and slug), inflammation-related proteins (e.g., Cox2, NF-*κ*B, and p53), and apoptotic molecules (Bax, Bcl-2) were found to drive cells through the inverse process, namely, MET upon therapy. Hence, in triple-negative BC, RSVL can positively potentiate SAL [93].

#### 3.1.2. Digestive Tract Cancers

*(1) HCC*. In HCC, quantitative proteomic analysis was employed to study EMT and associated molecular events. When activating HCC cell lines (HepG2 and Huh7), an increased EMT profile was observed. But when introducing also metformin, EMT and metastasis were inhibited, and moreover, metformin could inhibit AKT/GSK-3*β* signalling induced by bFGF-mediated activation [94]. Another process studied in HCC was inflammation, knowing that an inflammatory status promotes tumourigenesis [95]. In a mouse model, label-free quantitative (LFQ) proteomics was performed for protein identification during the transformation of hepatic cells subjected to an inflammatory milieu to a precancerous cellular pattern. In this process, several proteins were identified as being deregulated from integrin, Rho family GTPases, IL-8, and ILK signalling pathways. Deregulations in the processes that regulate focal adhesion and actin cytoskeleton were as well found. Western blot has shown that proteins in EMT were upregulated (e.g., p-STAT3, TWIST, SNAIL, vimentin, and MMP-9). This study demonstrated some new therapy targets in HCC from the inflammatory-related pathways activating EMT [96]. MMPs as proteins related to EMT process were reported in several other cancers, like melanoma [97], nonmelanoma [98, 99], and other skin-related tumours [100, 101], and modulated through therapy induction.

*(2) CRC*. In CRC cell lines, it was shown that cyclic AMP (cAMP) response element binding protein 1 (CREB1) can be a new therapy target. Luteolin was tested in cell lines, and it inhibited CREB1 expression blocking therefore EMT. Moreover, luteolin induced MET process and reduced EMT-protein expressions inhibiting tumour cell migration. When comparing proteomic profile of CRC cell lines, HCT-116, treated or not with luteolin, a large panel of proteins, over 360 proteins, with different expressions were identified. Immunoblot evaluation has shown that CREB1 protein is decreased upon luteolin treatment and that this expression is inhibited at the transcriptional level. Besides the knowledge presented in this study, the authors point out that “proteomics is a powerful platform” to be used in deciphering the mechanism of action for drugs that can target EMT [102].

#### 3.1.3. Lung Cancer

In the inflammatory environment of tumours, macrophages have an M2-phenotype that is protumourigenic. Zhu et al. showed that in lung cancer, M2 macrophages secrete factors that induce tumour cell migration, invasion, and EMT. In various cancers, *β*-elemene was shown to inhibit tumour proliferation [103]. In lung cancers, it was shown that *β*-elemene switched the polarization of protumourigenic M2-macrophages to M1. Moreover, this natural-related compound inhibited EMT characteristics of lung cancer cells and increased their radiosensitivity [104].

#### 3.1.4. Urinary Tract Cancers

In prostate cancer (PC), the effects of plectranthoic acid (PA), extracted from natural sources, were investigated on EMT, migration, and invasion in PC cell lines. The study showed that PA reversed EMT and the proteomic analysis has pinpointed Rac1 as significantly inhibited upon treatment, indicating thus PA as a new adjuvant drug in PC [105]. Another natural product, caffeic acid phenethyl ester (CAPE), was studied as a future advent drug in PC. In PC-3 and DU-145 prostate cancer cell lines using several proteomic technologies (gelatin zymography, Western blotting), EMT-related proteins were investigated upon CAPE treatment, and it was shown that MMP-9 and MMP-2 have reduced activities. Micro-Western Array, a proteomic platform, performed on this treated cell lines has shown that the compound reduced *β*-catenin abundance, NF-*κ*B activity, PI3K-Akt signalling, and EMT. In a mouse model of induced tumours, the immunohistochemical analysis has shown that the treatment increased ROR2 and Wnt5a expression and decreased Ki67, Frizzle 4, NF-*κ*B p65, MMP-9, Snail, *β*-catenin, and phosphorylation of I*κ*B*α* protein expression. In this study, proteomic technologies could pinpoint a new drug in PC that hinders aggressiveness and EMT processes [106].

In RCC, slightly different results were reported. Thus, using proteomic-based analyses, it was shown that HDACi do not induce EMT, contrarily inducing reduced proliferation and apoptosis. HDACi reduced E-cadherin and platelet-derived growth factor receptor-*β* (PDGFR*β*), reducing RCC metastatic capacity. Moreover, in animal models, lung metastasis was reduced by HDACi [107].

#### 3.1.5. Neurodegenerative Disorders

Histone deacetylase (HDAC) inhibitors have a long history of drugs, generally in neurodegenerative disorders [108], but recently, these drugs were tested also in oncology. In UC cell lines (RT112, VM-Cub-1, SW1710, and UM-UC-3), proteome and transcriptome investigations showed that HDAC5 overexpression in one of the investigated cell lines induced a decrease in cell proliferation but a switch to an EMT. This study signals proteomic mechanisms that are more subtle and more individualized in various tumour cell types as prior acknowledged [109].

In the neuroblastoma mouse model, the proteomic profile of murine anchorage-dependent (AD) cells and anchorage-independent (AI) tumour spheres was reported. Through complex proteomics, it was shown that there are upregulated proteins from the tumourigenic pathways in AI tumour spheres compared to AD cells. These proteins were regulating metastatic and EMT-related pathways. Thus, more specifically, survivin, CDC2, and the enzyme poly (ADP-ribose) polymerase 1 were found upregulated. The approved drug, sunitinib, inhibited PDGFR*β*, MYCN, and SOX2, and survivin in AI reduced tumour sphere self-renewal. Moreover, the radiosensitivity of AI was enhanced upon treatment. In neuroblastoma, there is a high heterogeneity of tumour cells, so all cell types should be targeted to have an overall efficient therapy [110].

### 3.2. Therapy Resistance with EMT Traits

Resistance to chemotherapy is a widely spread process along cancer. In this resistance process, EMT can induce resistance to cancer therapies, as proven throughout multiple types of cancers [111].

#### 3.2.1. Breast and Digestive Cancers

In CRC patients, recurrence after chemotherapy is probably due to CSCs. Using a yeast-2-hybrid system and 2D gel-based proteomics, recently, it was shown that there is a molecular link between CSC and EMT. Thus, E3-ubiquitin ligase FBXW7 binds to EMT-inducing transcription factor ZEB2 and further induces its degradation. The FBXW7-ZEB2 tandem regulates many cancer cell features like stemness, chemoresistance, and metastasis. An increased expression of ZEB2 in tumours is correlated with reduced ZEB2 expression in cancer-associated stroma sustaining a tumour-stromal crosstalk and EMT activation [112]. In CRC, the chemoresistance is associated with TP53 gene mutation. Investigating the mechanism of EMT associated to chemoresistance, EFNB2 was found as mutant p53 target responsible for chemoresistance. The acetylated version of mutant p53 protein is enlisted on the EFNB2 promoter and upregulates its expression in conjunction with coactivator p300. Silencing EFNB2 induces chemosensitivity in tumours that display mutant p53. Patients that had tumours with high expression of EFNB2 had low clinical response to neoadjuvant therapy. Targeting the ephrin-B2 axes can increase the therapeutic capacity of cytostatics in tumours that display mutant p53 [113]. In HCC, quantitative proteomics and phosphoproteomics were performed on metastatic tissues resistant to sorafenib treatment. Analysis has shown that there are overexpressed pathways involved in tumour progression and resistance, (e.g., EMT and cell adhesion). Using this proteomic protocol, mechanisms of resistance based on EMT processes can be elucidated and further can be developed through precision medicine [114].

In HER2-positive breast and gastric cancer, resistance to trastuzumab installs during disease evolution. Liu et al. established a set of cell lines (trastuzumab-resistant MKN45, NCI N87 gastric cancer sublines) in order to study the underlining resistance mechanisms. Using label-free quantitative proteomics, altered pathways related to EMT process were found in MKN45/R cells, the key altered pathway being Wnt/*β*-catenin. Using Western blot, proteins like Wnt3A, FZD6, and CTNNB1 were confirmed as increased, while GSK-3*β* was found decreased. Using a specific Wnt/&-catenin inhibitor in these cells, proliferation was reduced and EMT was reversed. The authors conclude that Wnt/*β*-catenin pathway sustains trastuzumab resistance, and using Wnt/*β*-catenin inhibitors can overcome the resistance in this case [115]. For investigating resistance in BC HER2-positive breast cancer, cell lines were generated. This cell line was resistant to either lapatinib or AZD8931, and resistance was associated to their EMT phenotype. A global proteomic methodology was applied to these cells, and a novel set of EMT-related proteins was identified. Targeting EMT-associated kinases (e.g., Src and Axl) inhibited cell proliferation thus providing an additional therapy option [116].

EMT association with chemoresistance in BC focused on PD-L1 expression was also studied. In a complex transcriptomic and proteomic endeavour, expression profiling for over 500 patients was performed. A positive correlation between PD-L1 expression and stemness score of the cancer tissue was found. Global proteomic analysis showed that AKT has a central role in PD-L1 expression. Downregulation of PD-L1 expression reduced the cancer cell stemness and EMT characteristics and brings new backgrounds for anti-PD-L1 therapy in BC [117].

Breast cancer cells (cell line MDA-MB-231) treated with metapristone reduce metastasis and their interference with endothelial cells in the process of migration. Using iTRAQ technology to assess the effect of metapristone on MDA-MB-231 cells, over 5,000 proteins were identified, out of which over 300 proteins were differently expressed in treated cells. These proteins were involved in seminal cellular processes, like translation, transcription, replication, and signal transduction. Proteins like E-cadherin, vimentin, TGF-*β* receptor I/II, smad2/3, *β*-catenin, caveolin, and dystroglycan are associated with signalling pathways (e.g., TGF-*β* and Wnt) linked to EMT process. Using immunoblot and immunofluorescence, the validation of EMT-related proteins (E-cadherin, vimentin) was done [118].

#### 3.2.2. Melanoma

In BRAFV600E-mutated melanoma tumours, the acquired resistance to MEK1/2 inhibitors (MEKi) reinstalls ERK1/2 signalling. In contrast, resistance installed by KRASG13D amplification drives ZEB1-dependent EMT and chemoresistance [119]. In melanoma patients, resistance to new therapy occurs due to the high plasticity and heterogeneity of this skin cancer. Several proteomic studies were focused on MAPK inhibitor (MAPKi) resistance. These studies have pointed out that BRAFi resistance is associated with lysosomal compartment, cell adhesion, and EMT process. When switching to the invasive state, melanoma cells gain EMT characteristics and become resistant to MAPKi. The authors point out that proteomic studies can further reveal other subtle mechanisms underlying MAPKi resistance mechanisms and develop biomarkers for identifying early resistance pattern [120, 121].

#### 3.2.3. Non-Small-Cell Lung Cancer

Drug resistance in non-small-cell lung cancer (NSCLC) was studied by evaluating proteomic profiling in a resistant A549 CDDP-resistant (CPr-A549) cell line. A panel of 15 proteins was found differentially expressed in CPr-A549. These proteins are involved in misfolding of proteins, endoplasmic reticulum stress, positively correlated with EMT and CSC markers. These proteins could be further developed in NSCLC patient prognosis and survival markers [122].

In NSCLC, the resistance to tyrosine kinase inhibitors (TKIs) is also related to the EMT process and CAFs. In experimental models with cell lines like HCC827 and PC9 cells, the interaction with CAFs induced EMT phenotype and overexpression of specific EMT markers. All these recent data were obtained using proteomic-based methods. Annexin 2, a protein influencing mainly the cellular motility [123], was found significantly increased on NSCLC cells by CAFs along with an increased secretion of growth factors HGF and IGF-1. Knocking down ANXA2 induced an inverse EMT phenotype. Thus, if a new therapeutical approach is to be designed in the future, inhibition of tandem HGF/c-met and IGF-1/IGF-1R networks could reduce EMT and gefitinib resistance [124].

#### 3.2.4. Radioresistance in Solid Tumours

Tumour resistance to radiotherapy is another challenge in tumour therapies. In PC, metastasis and recurrence postradiotherapy can occur. In an animal model, using a radioresistant xenograft mouse, pathways that can induce this resistance were investigated. Xenografts that were resistant in comparison to nonresistant cells (PC-3RR compared to PC-3) were analysed using liquid chromatography tandem-MS. There were reported almost 380 proteins and over 50 pathways that were significantly differently expressed in PC-3RR compared to PC-3 xenograft and that the deregulated glycolysis pathway links to PC radioresistance. Within the glycolysis pathway, lactate dehydrogenase A enzyme (LDHA) is a crucial node, and if knocked down (e.g., with siRNA or with a LDHA specific inhibitor like FX-11), PC-3RR cells would develop radiosensitivity, reduced EMT phenotype, hypoxia, and apoptosis [125, 126].

#### 3.2.5. Other Therapy Resistance Inducers

Associated with various cancer types, Nogo-B receptor (NgBR), was found highly expressed in BC, lung cancer, HCC, and so on. It seems that in all these types of cancer NgBR promotes EMT. Dong et al. have shown that in Bel7402/5FU cells, increased expression of NgBR is associated with chemoresistance. If this receptor is knocked down in this cell line, proteins p53 and p21 are reduced. NgBR expression in HCC is correlated with a poor prognosis. Thus, targeting NgBR and combining with chemotherapy (e.g., 5-FU) an increased therapeutic efficacy can be obtained [125]. In lung cancer, NgBR was studied as EMT inducer. Indeed, the NgBR knockdown of NSCLC cells inhibited EMT process, while NgBR overexpression induced EMT, through EMT-related proteins, mainly Snail1, the transcription factor repressing E-cadherin expression. NgBR overexpression favoured Ras membrane localization and the activation of MEK/ERK signalling. These results can aid to developing new therapeutic strategy in NSCLC [127].

In several tumour cell types, high EMT state and therapy resistance depend on the lipid-peroxidase pathway. This pathway activation protects tumour cell from ferroptosis. Using proteomic technologies, the enzymes that govern this process were identified. The lipid metabolism specific for tumour cells is dependent on phospholipid glutathione peroxidase (GPX4), an enzyme that prevents ferroptotic cell death. Therapy-resistant tumour cells have high EMT pattern and high expression of ZEB1. This configuration was proven in carcinomas, in TGF*β*-resistance in melanoma, and in treatment-induced transdifferentiation in PC and sarcomas. If this abnormal lipid peroxidase pathway can be targeted and reversed, then EMT-driven therapy resistance can be eluded [111].

An outline of proteomic technologies used for identification of EMT-related molecules in various cancers is presented in Table 1.

Nevertheless, it should be highlighted that proteomic approaches emerge in the EMT field by preparing the clinical setting ground because there are already plentiful preclinical approaches in this domain. There are *omic* platforms such as protein microarrays and/or XMAP arrays that provide besides multiplexing qualitative and quantitative detection for discovery and validation of EMT-related markers. Subtle molecular modifications are detected with multidimensional-electrophoresis and MS platforms that could discover and identify specific biomolecules of EMT trait. With the constant evolution of proteomic analysis, deficiencies associated with these techniques can hardly be stated. However, an important shortage related to omic platforms to be implemented in clinics could be linked to high equipment costs and way above medium personnel expertise. Nevertheless, protein microarrays open the clinical setting by designing special formats for diagnosis/early diagnosis or therapy monitoring [128]. A small volume of analysed samples, simultaneous detection and short operating time, and increased specificity and sensibility of the tested analytes sustain the importance of proteomic technologies in any biomedical attempt EMT process included [128, 129].

## 4. Conclusion

EMT is involved in key steps of tumour progression, because it mainly facilitates metastasis. Recently, EMT research has taken on board proteomic technologies to identify new molecular insights of the process and to further evaluate the possibilities to develop new therapy targets. Various proteomic tools and multiple combinations were developed in this area. Out of the proteomic technology armentarium, mass spectrometry and array technologies are the most used approaches [129]. The main characteristics of the proteomic technology used in this domain are high throughput and detection of minute concentration in small samples. Therefore, in various cancer cell lines and tumour tissues, EMT-related proteins were newly detected proteins that are involved in the activation of different cellular pathways. Proteomics has brought besides standard EMT markers (e.g., cell-cell adhesion proteins and transcription factors) other future potential markers for improving diagnosis, monitoring evolution, and developing new therapy targets. Thus, proteomics will steadily increase its role in clinical investigation and validation of EMT-related biomarkers [24].

## Figures and Tables

**Table 1:** Proteomic platforms for EMT process evaluation in various types of cancer tissues.

Proteomic platform(s) in EMT assessing	Cancer type/sample type	Evaluated EMT-related process	Comments regarding EMT-related proteins	Ref
iTRAQ-based quantitative proteomics	Colorectal cancer/tumour tissue	Tumourigenesis	TME proteins—ECM-receptors, focal adhesion, PI3K-Akt signalling pathway, angiogenesis, HIF-1 signalling pathways	[53, 54]
Reverse phase protein array	Colorectal cancer/tumour tissue	Recurrence	Collagen VI, FOXO3a, INPP4B, LcK, phospho-PEA15, phospho-PRAS40, Rad51, phospho-S6	[56]
Tissue microarray	Colorectal cancer/tumour tissue	Metastasis	Cdc42BPA highly correlated with metastasis	[65]
Yeast-2-hybrid system and 2D gel-based proteomics	Colorectal cancer	Therapy resistance	FBXW7-ZEB2 tandem involved in stemness, chemoresistance, metastasis	[112]
Two-dimensional fluorescence gel electrophoresis	Hepatocellular carcinoma	Metastasis	GnT-V-mediated N-glycosylation of marker CD147/basigin. Upregulated and controlled by PI3K/Akt pathway	[68]
Label-free quantitative proteomics	Hepatocellular carcinoma	Inflammation	Inflammatory milieu has integrin, Rho family GTPases, IL-8, and ILK signalling pathway deregulations	[96]
Quantitative proteomic analysis	Hepatocellular carcinoma	Therapy resistance	Metformin inhibits AKT/GSK-3*β* signalling	[94]
Quantitative (phospho)proteomics	Hepatocellular carcinoma resistance	Therapy resistance	Proteins related to EMT and cell adhesion were associated with sorafenib treatment resistance	[114]
Label-free quantitative proteomics	Gastric cancer	Therapy resistance	Wnt/*β*-catenin pathway sustains trastuzumab resistance	[115]
Micro-Western Array	Prostate cancer	Therapy resistance	Caffeic acid phenethyl ester reduced *β*-catenin, NF-*κ*B, and PI3K-Akt signalling	[106]
Liquid chromatography tandem-mass spectrometry	Prostate cancer	Therapy resistance	Signalling pathways in therapy-resistant tumours	[126]
Liquid chromatography-selected reaction monitoring mass spectrometry	Breast cancer	Aggressiveness	Estrogen, progesterone receptors, HER2/Neu receptor correlated with Ki-67 and vimentin	[72]
2D-differential gel electrophoresis, MALDI-MS, immunoblotting	Breast cancer	Aggressiveness	ECM pattern—fibrinogen-*β* chain, collagen *α*-1(VI) chain, and *α*-1B-glycoprotein	[73]
Flow cytometric surface proteomics	Breast cancer	Aggressiveness	Upregulated proteins CD200, CD51/CD61, CD26, CD165, and CD54	[76]
Reverse phase protein arrays	Breast cancer	Subtyping	Immune-related subtype and a hormone-related subtype	[78]
Quantitative multiplexed proteomic tandem mass tags	Breast cancer	Therapy resistance	TACC3 inhibitor, suppresses cancer cell stemness	[91]
Western blot, colony formation, flow cytometry for cellular apoptosis	Breast cancer	Therapy resistance	Resveratrol and salinomycin reverse EMT	[93]
iTRAQ technology	Breast cancer	Therapy resistance	E-cadherin, vimentin, TGF-*β* receptor I/II, smad2/3, *β*-catenin, caveolin, dystroglycan overexpression	[118]
Mass spectrometry	High-grade serous ovarian cancer	Tumourigenesis	Alpha-enolase and vimentin overexpression	[80]
GEL-LC-MS/MS and SILAC	Ovarian adenocarcinoma	Tumourigenesis	Activated signalling pathways PI3K/Akt/mTOR and Ras/Erk MAPK	[81]
Mass spectrometry-based quantitative proteomic approaches	Cervical cancer	Tumourigenesis	Myoferlin regulates ADAM12 expression	[83]
Membrane proteomic methodology	Head and neck squamous cell carcinoma	Tumourigenesis	EGFR constitutively phosphorylated	[85]
